# P-366. Clinical Experience of Long-Acting Cabotegravir/Rilpivirine in an Underserved Population in Houston, Texas

**DOI:** 10.1093/ofid/ofaf695.584

**Published:** 2026-01-11

**Authors:** Livia Frost, Shital M Patel, Thomas P Giordano, Melanie C Goebel, Onachi Isiofia, Guillermo Ernesto Linares Pineda

**Affiliations:** Baylor College Of Medicine, Houston, TX; Baylor College of Medicine, Houston, Texas; Baylor College of Medicine, Houston, Texas; Baylor College of Medicine, Houston, Texas; Baylor College of Medicine, Houston, Texas; Baylor College of Medicine, Houston, Texas

## Abstract

**Background:**

Oral antiretroviral therapy (ART) has made HIV a manageable chronic condition, however, adherence challenges persist. LA-CAB/RPV offers an ART alternative. Clinical trials demonstrate high patient satisfaction and efficacy of LA-CAB/RPV, however, data is limited on real-world experiences among people with HIV (PWH) in the U.S. South, especially in underserved populations.

**Figure:**
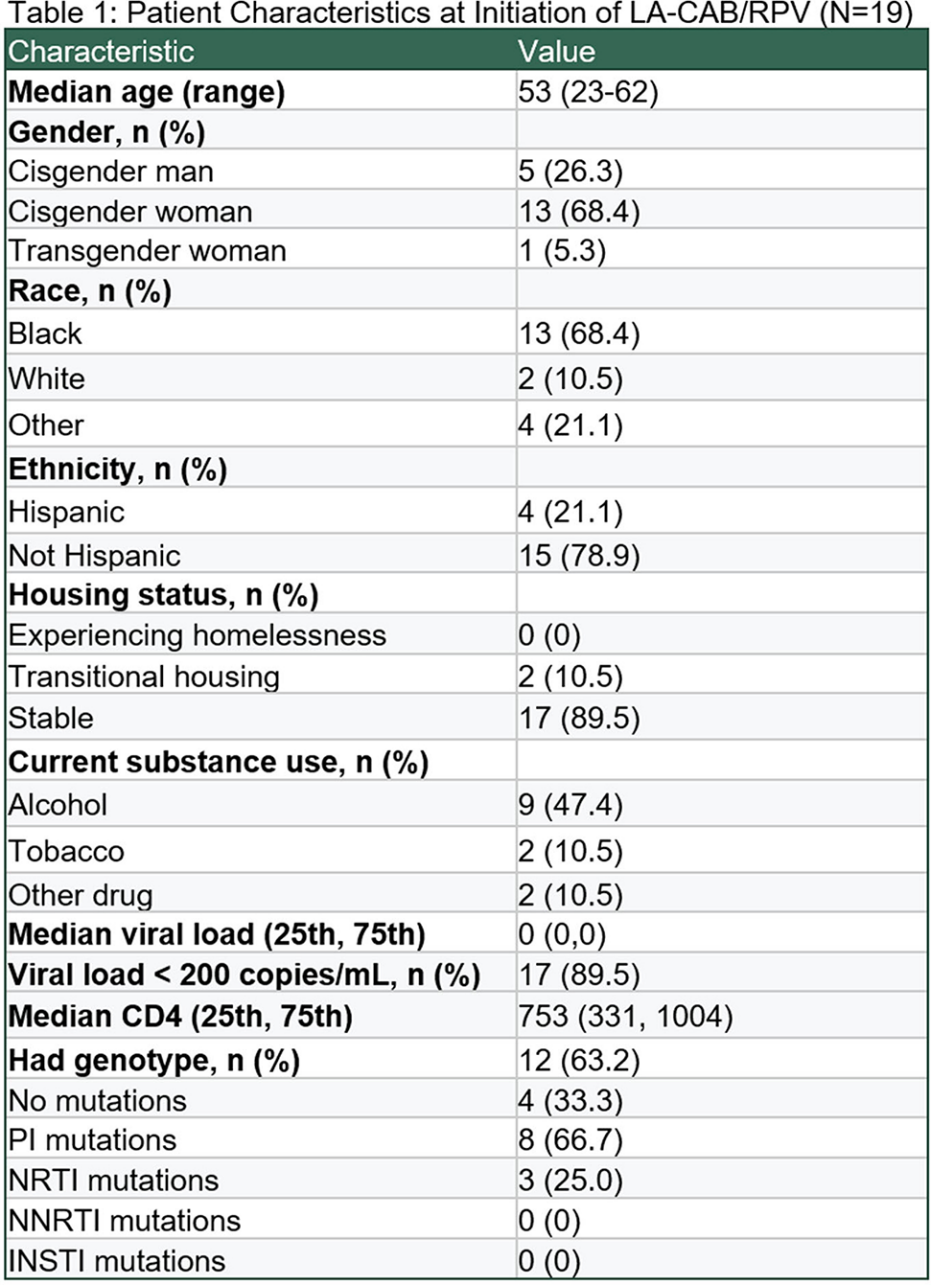

**Figure 1.**
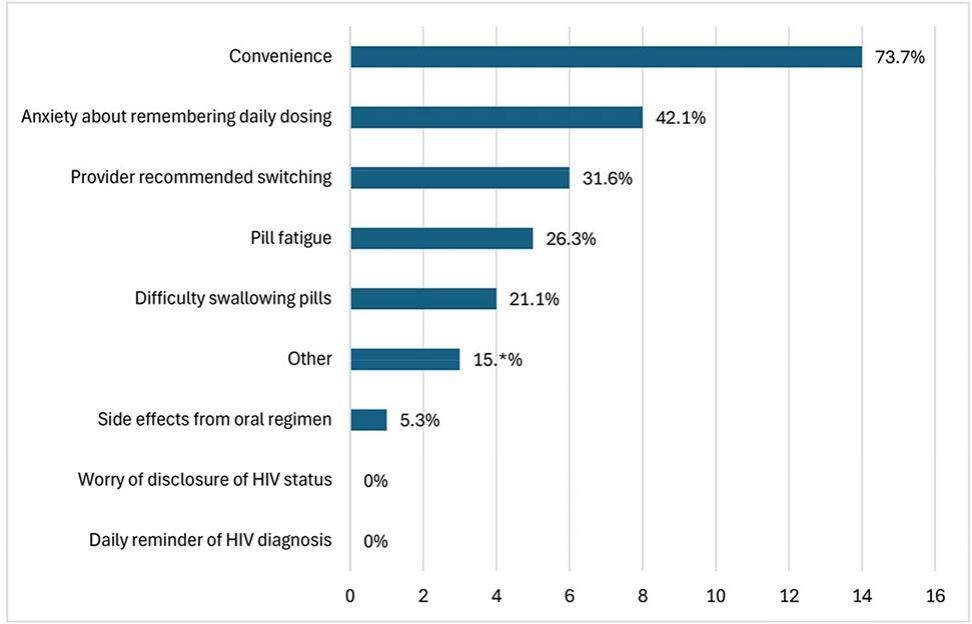
Participant-reported reason for switching from oral ART to LA-CAB/RPV (N=19)

**Methods:**

A prospective cohort of PWH who received at least one dose of LA-CAB/RPV was established at a Ryan White-funded clinic in Houston, Texas. Participants completed a survey on demographics and experience with LA-CAB/RPV. Clinical and laboratory data were collected from the medical record.

**Figure 2.**
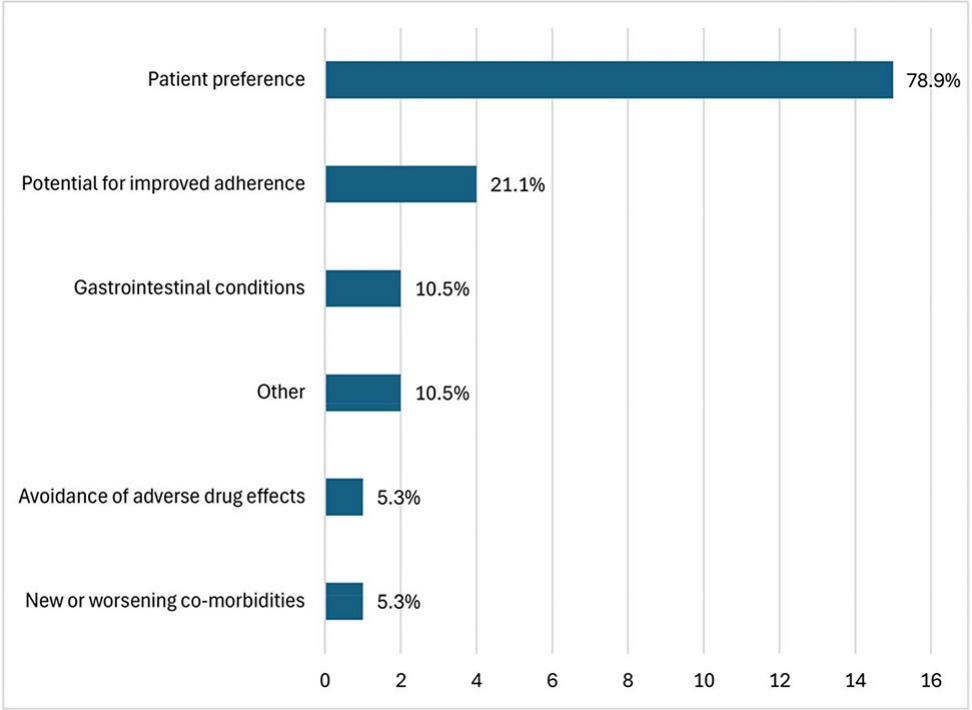
Provider-reported reason for switching from oral ART to LA-CAB/RPV (N=19)

**Figure 3:**
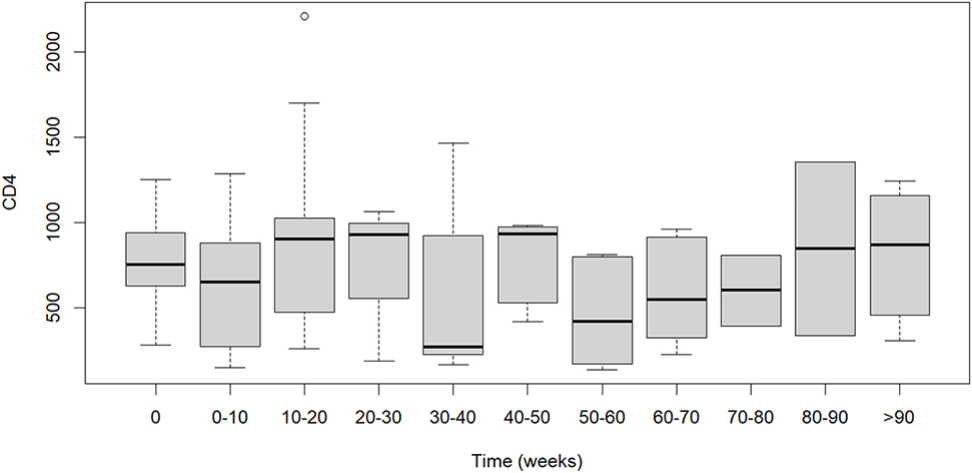
CD4 cell counts (x109 cells/L) following LA-CAB/RPV initiation (Time 0 = first injection of LA-CAB/RPV) (N=19)

**Results:**

Currently, 19 participants are enrolled (21.1% Hispanic, 68.4% Black, 68.4% cisgender female). The median age is 53 years, and the median time since HIV diagnosis is 16 years. Patient-reported reasons for switching from oral ART to LA-CAB/RPV included convenience (73.7%), anxiety about daily dosing (42.1%), provider recommendation (31.6%), pill fatigue (26.3%), difficulty swallowing pills (21.1%), and side effects from oral ART (5.3%). Provider-reported reasons for switching to LA-CAB/RPV included patient preference (78.9%), potential for improved adherence (21.1%), and gastrointestinal conditions (10.5%). Common side effects included injection site pain (63.6%) and swelling (27.3%). Two participants discontinued LA-CAB/RPV due to intolerance of injections. 17 of 19 (89%) participants had VL< 50 c/mL at the time of switching to LA-CAB/RPV. Two of these participants discontinued LA-CAB/RPV due to intolerance of injections, with VL< 50 c/mL, and the remaining 15 participants maintained VL suppression (mean follow up 69.5 weeks). 2 patients had VL > 200 c/mL (3,682 and 40,600) at the time of switch, and both achieved virologic suppression on LA-CAB/RPV. CD4 counts were stable for all participants after switching.

**Conclusion:**

In an underserved population of PWH in the US South, LA-CAB/RPV offers a patient-centered alternative to oral ART, with patient preference being the most common reason for switching to injectable ART. LA-CAB/RPV was effective, with high rates of virologic suppression, including among 2 patients with viremia at the time of switching.

**Disclosures:**

All Authors: No reported disclosures

